# Prevalence, types and determinants of anemia among pregnant women in Sudan: a systematic review and meta-analysis

**DOI:** 10.1186/s12878-018-0124-1

**Published:** 2018-11-08

**Authors:** Ishag Adam, Yassin Ibrahim, Osama Elhardello

**Affiliations:** 1 Faculty of Medicine, University of Khartoum, P.O. Box 102, Khartoum, Sudan; 2 Faculty of Medicine, University of Tabuk, P.O. Box 741, Tabuk, Saudi Arabia; 3 Scarborough General Hospital, Scarborough, YO12 6QL UK

**Keywords:** Prevalence of anemia, Anemia during pregnancy, Age, Parity, Malaria during pregnancy, Sudan, Meta-analysis, systematic review

## Abstract

**Background:**

Anemia during pregnancy is a public health problem especially in developing countries and it is associated with maternal and perinatal adverse outcomes. There is no meta-analysis on anemia during pregnancy in Sudan. The current systemic review and meta-analysis was conducted to assess the prevalence, types and determinant of anemia during pregnancy in Sudan.

**Methods:**

Preferred Reporting Items for Systematic Reviews and Meta-Analyses (PRISMA) guideline was followed. The databases (PubMed, Cochrane Library, Google Scholar, CINAHL, and African Journals Online) were searched using; anemia, pregnancy related anemia and Sudan. Joanna Briggs Institute Meta-Analysis of Statistics Assessment and Review Instrument (JBI-MAStARI) and Modified Newcastle – Ottawa quality assessment scale were used for critical appraisal of studies. The pooled Meta logistic regression was computed using OpenMeta Analyst software.

**Results:**

Sixteen cross-sectional studies included a total of 15, 688 pregnant women were analyzed. The pooled prevalence of anemia among pregnant women in Sudan was 53.0% (95%, CI = 45.9–60.1). The meta-analysis showed no statistical significant between the age (mean difference = 0.143, 95 CI = − 0.033 − 0.319, *P* = 0.112), parity (mean difference = 0.021, 95% CI = − 0.035 − 0.077, *P* = 0.465) between the anemic and no anemic women. Malaria was investigated in six studies. Pregnant women who had malaria infection during pregnancy were 1.94 times more likely to develop anemia than women who had no malaria infection (OR = 1.94, 95% CI =1.33–2.82). Six (37.5%) studies investigated type of anemia. The pooled prevalence of iron deficiency anemia (IDA) among pregnant women in Sudan was 13.6% (95% CI = 8.9–18.2).

**Conclusion:**

There is a high prevalence of anemia among pregnant in the different region of Sudan. While age and parity have no association with anemia, malaria infection was associated with anemia. Interventions to promote the strengthening of antenatal care, and access and adherence to nutrition, and malaria preventive measures are needed to reduce the high level of anemia among pregnant women in Sudan.

**Electronic supplementary material:**

The online version of this article (10.1186/s12878-018-0124-1) contains supplementary material, which is available to authorized users.

## Background

Anemia during pregnancy is a public health problem especially in developing countries and it is associated with maternal and perinatal adverse outcomes [1]. World Health Organization (WHO) has defined anemia in pregnancy as the hemoglobin concentration of less than 11 g/dl [2]. According to WHO, anemia is considered of a severe public health significance if its rate of ≥40% [3]. Global data shows that 56% of pregnant women in low and middle income countries have anemia [1]. The prevalence of anemia is highest among pregnant women in sub- Sahara Africa (57%), followed by pregnant women in South-East Asia (48%) and lowest prevalence (24.1%) was reported among pregnant women in South America [3].

The causes of anemia during pregnancy in developing countries are multifactorial; these include micronutrient deficiencies of iron, folate and vitamin A and B12, anemia due to parasitic infections such as malaria and hookworm or chronic infections like tuberculosis and HIV [4, 5]. Contributions of each of the factors that cause anemia during pregnancy vary due to geographical location, dietary practice and season. In Sub Saharan Africa inadequate intake of diets rich in iron is reported as the leading cause of anemia among pregnant women [4–6].

Anemia during pregnancy is reported to have negative maternal and child health effect and increase the risk of maternal and perinatal mortality [7, 8]. The negative health effects for the mother include fatigue, poor work capacity, impaired immune function, increased risk of cardiac diseases and mortality [1, 8]. Some studies have shown that anemia during pregnancy contributes to 23% of indirect causes of maternal deaths in developing countries [1]. Although, there many published studies on anemia during pregnancy in the different setting of Sudan [9–24], there is no systemic review/ meta-analysis on anemia in Sudan. The current systemic review and meta-analysis was conducted to assess prevalence, types and determinant of anemia during pregnancy in Sudan.

## Methods

### Study design and search strategy

Findings from published studies were used to conduct this systematic review and meta-analysis to determine the prevalence of anemia, types and its determinants (age, parity and malaria) among pregnant women in Sudan. The major databases of PubMed, Cochrane Library, Google Scholar, CINAHL, and African Journals Online were reviewed for all published studies relevant to anemia during pregnancy and its determinant factors.

All studies that were published up to April 03/2018 were retrieved to be assessed for eligibility of inclusion in this review. In addition, the reference list of each included study was also searched, retrieved and assessed for inclusion eligibility.

The terms that used for searching are: “anemia OR anemia during pregnancy OR determinants of anemia AND Sudan”. Preferred Reporting Items for Systematic Reviews and Meta-Analyses (PRISMA) guideline was followed for conduction of this systematic review and meta-analysis [25].

### Study selection and eligibility criteria

#### Outcome of interest

The primary outcome of this study was the prevalence of anemia during pregnancy. The WHO defines anemia in pregnancy as low blood hemoglobin concentration, below 11 g/dl or hematocrit level less than 33% dl [2]. The secondary outcomes were; types and determinants (age, parity and malaria) of anemia during pregnancy.

#### Quality assessment and data collection

The included studies were assessed by using Joanna Briggs Institute Meta-Analysis of Statistics Assessment and Review Instrument (JBI-MAStARI) [26]. Modified Newcastle – Ottawa quality assessment scale for cross sectional studies was used to assess the quality of the study for inclusion [27]. The total score for the modified Newcastle – Ottawa scale for cross sectional studies is nine (9) stars as a maximum for the overall scale with the minimum of zero. A study was considered high quality if it achieved 7 out 9 and medium if it achieved 5out of 9, Table 1, Additional file 1.

**Table 1:** Summary and the assessment of the included studies

The study	Year	Location	Sample size	Prevalence of anemia%	Score of the Modified Newcastle Ottawa Scale
Abas et al.,	2017	Khartoum	423	57.68	5
Abdelgadir et al.,	2012	Geizera	292	40.75	6
Abdelrahman	2012	Khartoum	194	24.23	6
Abdelrahim et al.,	2009	Gadarif	300	74.67	7
Abdullahi et al.,	2014	Khartoum	856	47.78	6
Adam a et al.,	2005	New Halfa	744	62.63	7
Adam b et al.,	2005	New Halfa	125	76	7
Adam c et al.,	2007	New Halfa	333	36.94	6
Adam d et al.,	2012	Geizera	324	62.04	7
Ali et al.,	2011	Kassala	9578	41.89	6
Bushra et al.,	2010	Geizera	200	52	7
Elmugabil et al.,	2017	Khartoum	338	49.7	6
Haggaz et al.,	2010	Darfur	403	74.69	6
Mohamed et al.,	2011	Kassala	250	58.4	6
Mubarak et al.,	2014	Khartoum	179	23.46	8

Two reviewers (IA & YI) independently assessed the quality of each article for inclusion in the review. The disagreement arise between the reviewers was resolved through discussion and involvement of a third reviewer (OE).

A tool for data extraction was developed to extract the most important relevant information for the review. It consists of tables that include information about the authors’ name, year of publication, study location, sample size, age of study participants, and number of pregnancies, malaria with pregnancy, type of anemia and presence and types of complications of anemia.

#### Data analysis and heterogeneity assessment

OpenMeta Analyst software for Windows [28, 29] was used to perform all the meta-analyses of prevalence and determinants (age, parity and malaria) of anemia. The heterogeneity of the included studies was evaluated using Cochrane Q and the I^2^. Cochrane Q with *P* < 0.10 and I^2^ > 50 was taken as standard to indicate the presence of heterogeneity of the included studies [30]. Based on the results of the analysis of Cochrane Q and I^2^ the random effects or fixed model was used to combine the included studies accordingly. A sub-group analysis was performed to investigate the association between malaria and anemia.

## Results

### Study selection

**Fig. 1:**
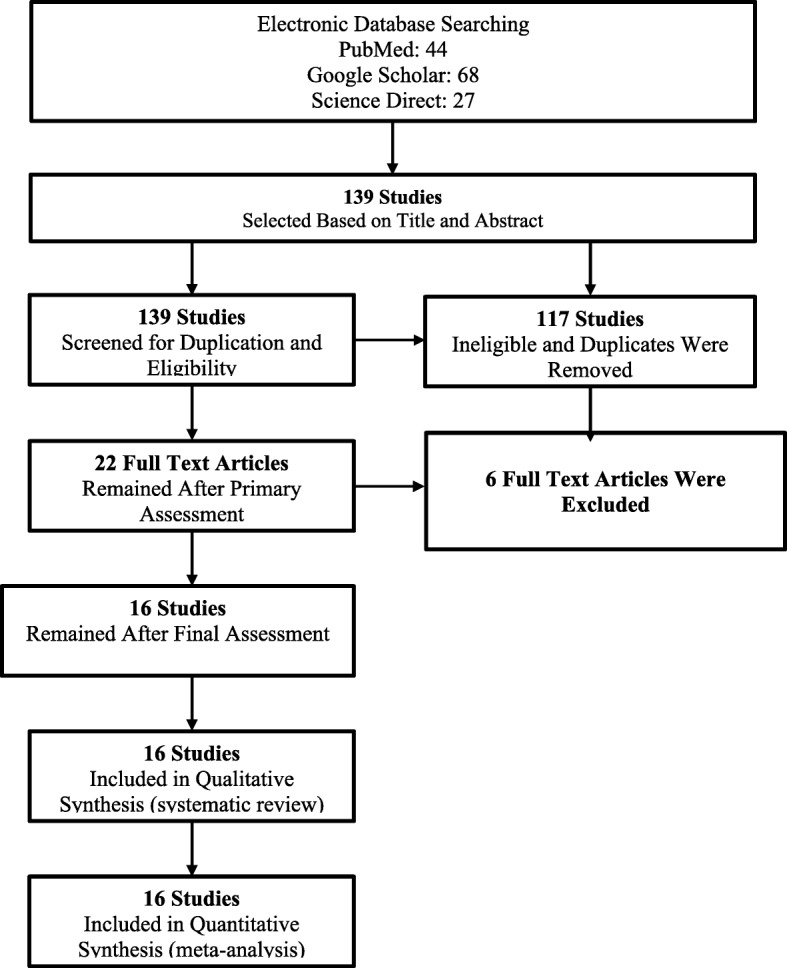
Flow diagram showing the number of articles identified in the systematic review and meta-analysis on anemia during pregnancy in Sudan

The reviewers found a total of 139 published articles initially. Out of these, 117 were removed due to duplicate records and not fulfilling the criteria. A total of 22 full-text articles were screened for eligibility. Out of these 22, 6 articles were excluded (using varied hemoglobin cut- off, case- control studies and included non-pregnant women. Sixteen studies were included in the final analysis, Fig. 1.

### Characteristics of included studies

Sixteen cross-sectional studies were included in this meta-analysis [9–24], Fig. 1. All of the included studies were conducted at health institutions, Additional file 2. All the studies used the WHO definition of anemia during pregnancy which is 11 g/dl [2].

Five (31.2%) of the 16 studies in this review were conducted in Khartoum [15, 17–19, 21], six (37.5%) studies were conducted in eastern Sudan (New Half, Kassala and Gadarif) [9, 10, 13, 22–24], three studies were conducted in Geizera [12, 14, 16], one study was conducted in Darfur and one study was conducted in Blue Nile [11, 20], Additional file 2.

The minimum sample size was 125 participants in a study conducted in New Halfa [22]. The higher sample size was 8578 conducted in Kassala in Eastern Sudan [24]. Overall, this meta-analysis included a total of 15, 688 pregnant women. The mean (SD) of the age and parity of pregnant women included in this review was 28.5 years and 2.08, respectively, Additional file 2. Six of 16 studies were conducted during delivery/labour (because investigating other outcome e.g. birth outcome), 2 studies were conducted in early pregnancy and the rest in the early third trimester, Additional file 2.

### Prevalence of anemia among pregnant women

**Fig. 2:**
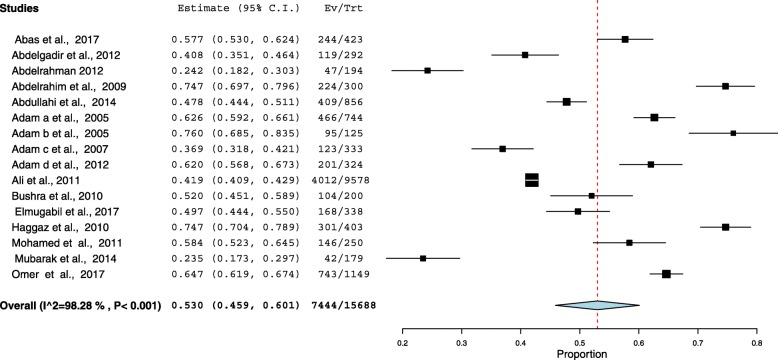
Forest Plot of the overall prevalence of anemia among pregnant women in Sudan

The minimum prevalence of anemia was 23.46% observed in a study conducted in Khartoum [15]. The highest, 76.0% was observed in a study conducted in Eastern Sudan [9]. The I^2^ test result showed high heterogeneity (I^2^ = 98.2%, *p* < 0.001). Using the random effect analysis, the pooled prevalence of anemia among pregnant women in Sudan was 53.0% (95% CI = 45.9–60.1), Fig. 2.

### Association between age, parity and anemia

**Fig. 3:**
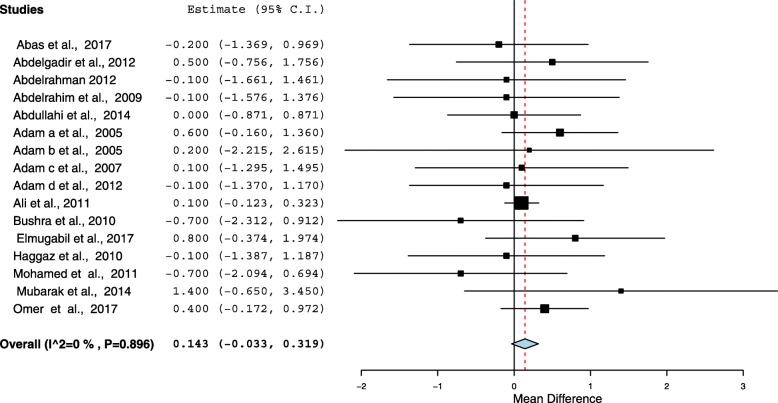
Forest Plot of the age difference in anemia and non-anemic pregnant women in Sudan

**Fig. 4:**
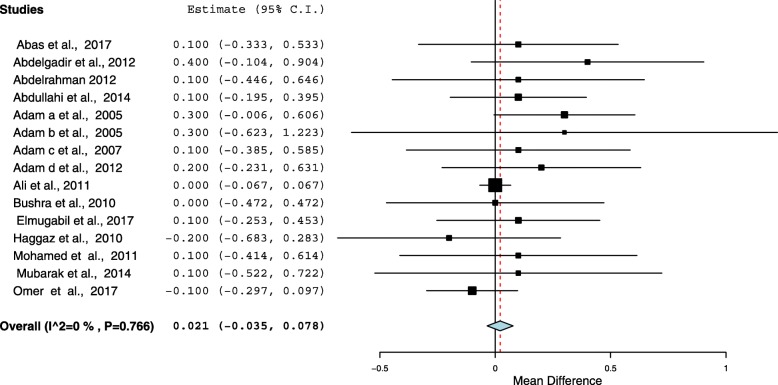
Forest Plot of the parity difference in anemia and non-anemic pregnant women in Sudan

All the included studies (16) reported the age and parity as continuous variables. Therefore the mean (SD) was compared in this review. The meta-analysis showed no statistical significant between the age (mean difference = 0.143, 95% CI = − 0.033 − 0.319, *P* = 0.112), parity (mean difference = 0.021, 95% CI = − 0.035 − 0.077, *P* = 0.465) between the anemic and no anemic women, Figs. 3 and 4. The heterogeneity test showed no statistical evidence of heterogeneity (*p* = 0.896), therefore the fixed model was used.

### Association between malaria infection and anemia

**Fig. 5:**
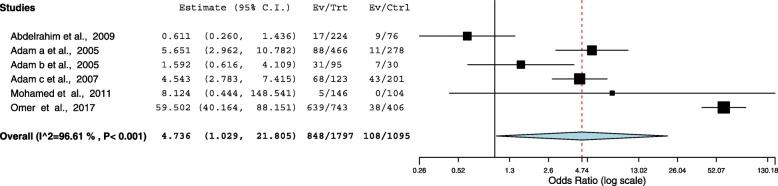
Forest Plot of the association between malaria and anemia in pregnant women in Sudan

Malaria was investigated (peripheral/ placental) in six studies [9, 10, 13, 20, 22, 23], Additional file 3. Pregnant women who had malaria infection during pregnancy were almost two times more likely to develop anemia during pregnancy than women had no such infection (OR = 1.94 (95% CI = 1.33–2.82). The heterogeneity test showed statistical evidence of heterogeneity, *p* = < 0.001. Therefore random-effects analysis was used, Fig. 5.

### Type of anemia and micronutrient deficiency

**Fig. 6:**
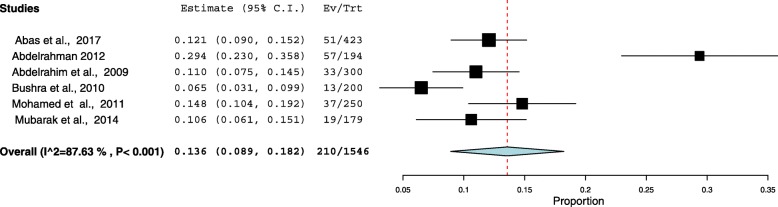
Forest Plot of the overall prevalence of iron deficiency anemia among pregnant women in Sudan

Six (37.5%) out of the 16 included studies investigated type of anemia [10, 12, 13, 15, 17, 19]. The minimum prevalence of iron deficiency anemia (IDA) was 6.5% observed in a study conducted in Geizera [12]. The highest prevalence of IDA was 29.3% which was observed in a study Khartoum [17], Additional file 1. The I^2^ test result showed high heterogeneity (I ^2^ 87.6%, *p* < 0.001). Using the random effect analysis, the pooled prevalence of IDA among pregnant women in Sudan was 13.6% (95% CI = 8.9–18.2), Fig. 6.

Two studies; in Geizera [12] and in Kassala [13] reported zinc deficiency rate of 45.0% and 38.0%, respectively. Only one study in Gadarif reported 57.7% and 1% of folate and B12 deficiency, respectively [10]. Only one study reported 4.0% copper deficiency in pregnant women in Geizera [12]. C-reactive protein was investigated in one study [13].

## Discussion

The main results of this met- analysis is the high prevalence (53.0%) of anemia among pregnant women in the different regions of Sudan. In neighbouring Ethiopia (meta-analysis of twenty studies and a total of 10, 281 pregnant women) the pooled prevalence of anemia among pregnant women in Ethiopia was 31.66% [31]. The prevalence of anemia in this meta-analyses is higher than the rate of anemia among pregnant women in the other African countries e.g. in Uganda 22.1%; [32]. A recent meta-analysis on global rate of anemia reported a lower rate (38%) of anemia in pregnant women, especially among pregnant women in East Africa where the prevalence of anemia was 36% [33]. This high prevalence of anemia in Sudan, is indicative of a severe public health problem, according to the WHO, anemia is considered a severe health problem if prevalence of anemia of ≥40% in a population [3]. It is worth to be mentioned that all the studies included in this meta-analyses were intuitional studies; therefore their results might not reflect the real situation in the community. Recently Kassa et al., have reported that the rate of anemia in pregnancy was much higher in the pooled meta-analyses (31.66%) that the rate (29.0%) of the anemia reported in the national (Ethiopia) survey [31, 34].

The current pooled meta-analyses showed that the age and parity were not different between anemic and non-anemic women. This reflects that anemia affects pregnant Sudanese women regardless to their age or parity. Recent meta-analyses showed that primigravidae were at lower risk for anemia compared with parous women [31]. Likewise, pregnant women with gravidity three to five and six and above were at 1.78, and 2.59 higher risks for anemia [35]. Wessells and colleauges have recently reported that gravidity and malaria were associated with associated with micronutrient deficiency status in pregnant women in Niger [36]. It is belived that women with high parity have low or no iron staorge as it hass been depleted by the repeated pregnaied hence parous women are more likely to be anemic [8]. Generally various risk factors (residence, parity, nutritional) for anemia during have been reported in Ethiopia [35, 37–41]. In Nigeria pregnant women in the rural communities had high pevalence of anemia and iron deficiency anemia compared to pregnant women in the urban setting [42]. In Ugandan Obai et al., have reported that lower prevalence of anemia (22.1%) among pregnant women and housewife were at 1.7 higher risk of anemia [32].

In the current meta-analysis 13.6% of among pregnant women in Sudan had IDA. This rate (13.6%) of IDA was higher than the rate (5%) of IDA reported among rural women of reproductive age in southern Ethiopia [43]. However, 20.7% of pregnant women in Niger had IDA [36].

The pathophysiological mechanism of anemia and its associations with malaria is not yet fully understood. Howver poor inake of food during illness, hemolysis, anemia of inflammation, bone marrow suppression, and micronutrients deficiency are the plasuible explnations for anemia and malaria [44].

### Limitiations

Some points need to be mentioned: fisrtly all of these studies were instituational ones. Thus the the findings of these studies might not indicate what is going at the community level. Secondly anemia during pregnancy in Sudan (included in these studies) has been studied mainly in Khartoum, Eastern Sudan and Geizera. There is a pauxity of reports on anemia during pregnancy in the other regions of Sudan e.g.White Nile, North Sudan and Khordofan. Thirdly, types and predictors for anemia during pregnancy were not deeply investiagted in these studies. Some factors such as antenatal care, food security, dietary diversity, maternal education and income need to be taken in the future research. Other types and causes of anemia e.g. folate and vitamin B12 deficiency need to be addressed in more depth.

## Conclusion

There is a high prevalence of anemia among pregnant in the different region of Sudan. While age and parity have no association with anemia, malaria infection was associated with anemia. Interventions to promote the strengthening of antenatal care, and access and adherence to nutrition, and malaria preventive measures are needed to reduce the high level of anemia among pregnant women in Sudan.

## Additional files

Additional file 1: Assessment of publication bias. (XLSX 13 kb)

Additional file 2: Characteristics of all studies included in the systematic review and meta-analysis. (XLSX 13 kb)

Additional file 3: Characteristics of the studies investigated malaria and anemia. (XLSX 10 kb)

## Abbreviations

CI: Confidence interval
IDA: Iron deficiency anemia
OR: Odd ratio
WHO: World Health Organization
